# A Novel Jak1 Gene Mutation in Invasive Breast Carcinoma

**DOI:** 10.1111/jcmm.70894

**Published:** 2025-10-26

**Authors:** Paras Kumar, Monika Rajput, Manoj Pandey

**Affiliations:** ^1^ Department of Surgical Oncology Institute of Medical Sciences, Banaras Hindu University Varanasi India; ^2^ DHR‐ICMR Advanced Molecular Oncology Diagnostic Services (DIAMOnDS) Institute of Medical Sciences, Banaras Hindu University Varanasi India

Dear Editor,

Breast cancer is the most prevalent cancer among women worldwide, accounting for 25% of cancer cases and 15% of cancer‐related deaths [1]. Its incidence is rising in developing countries. High‐penetrance mutations in BRCA1, BRCA2, TP53, and CHEK2 significantly elevate breast cancer risk, with BRCA mutations conferring a lifetime risk of 26%–85% [2]. Approximately 11% of families with multiple breast cancer cases under age 60 carry CHEK2 mutations [3, 4]. Next‐generation sequencing (NGS) has expanded the identification of breast cancer‐associated genes, including low‐penetrance variants like FGFR2, LSP1, and MAP3K1 [5]. JAK1, a protein tyrosine kinase gene on chromosome 1p31.3, is involved in the JAK/STAT signalling pathway and is altered in 1.88% of cancers, including IDC [6]. We describe here a novel JAK1 mutation in a 53‐year‐old woman with IDC.

A 53‐year‐old hypothyroid woman, on thyroxine, presented with a 3 × 2 cm lump in the upper outer quadrant of her right breast and mobile axillary lymph nodes. Core needle biopsy confirmed IDC (not otherwise specified, NOS), clinical stage cT2N1M0, oestrogen/progesterone receptor‐positive (ER/PR+), HER2‐negative, with a Ki67 of 5%–10%. She received four cycles of neoadjuvant Adriamycin and Cyclophosphamide, followed by breast conservation surgery and four cycles of adjuvant Docetaxel. Postoperative histopathology revealed IDC (ypT1cN0, Grade I) with a 15 mm ductal carcinoma in situ (DCIS) focus, and no lymphovascular invasion. NGS analysis identified a rare pathogenic JAK1 mutation (c.2554+1G>A, intron 18, chromosome 1:g.65307133 C>T) with a 5% mutant allelic burden and a splice donor single nucleotide variant (SNV). The tumour had low tumour mutation burden (TMB) and was microsatellite stable (MSS). Variants of uncertain significance (VUS) were noted in APC (c.4856C>T, p.Pro1619Leu) and TNFAIP3 (c.1054G>A, p.Glu352Lys). No alterations were found in BRCA1, BRCA2, TP53, PTEN, or other tested genes. After radiation therapy, she began anastrozole (1 mg daily) for 5 years. Five years post‐diagnosis, she remains disease‐free and is off hormone therapy.

JAK1, a dual oncogene and tumour suppressor, mediates IL‐6 cytokine signalling via the JAK/STAT pathway, influencing cancer development and immune homeostasis [7]. In breast cancer, ERBB2 signalling drives STAT3 activation through JAK1, promoting oncogenesis [8]. The novel JAK1 mutation (c.2554+1G>A) disrupts the splice donor site at exon‐intron 18, likely causing premature protein truncation, loss of pseudokinase and catalytic domains, and impaired JAK1 function (Figure 1). This may facilitate tumour immune evasion by reducing JAK1‐mediated interferon responses [9]. In silico tools (SpliceAI, score 0.99; VarSome) classified this variant as likely pathogenic, unreported in 1000 Genomes, gnomAD, or ClinVar, confirming its novelty. Further testing on PredictSNP version 2.1 showed deletion in splice function [10] while Mutation Taster showed this single nucleotide change as deleterious, leading to splice donor loss (Supporting information) [11].

**FIGURE 1:**
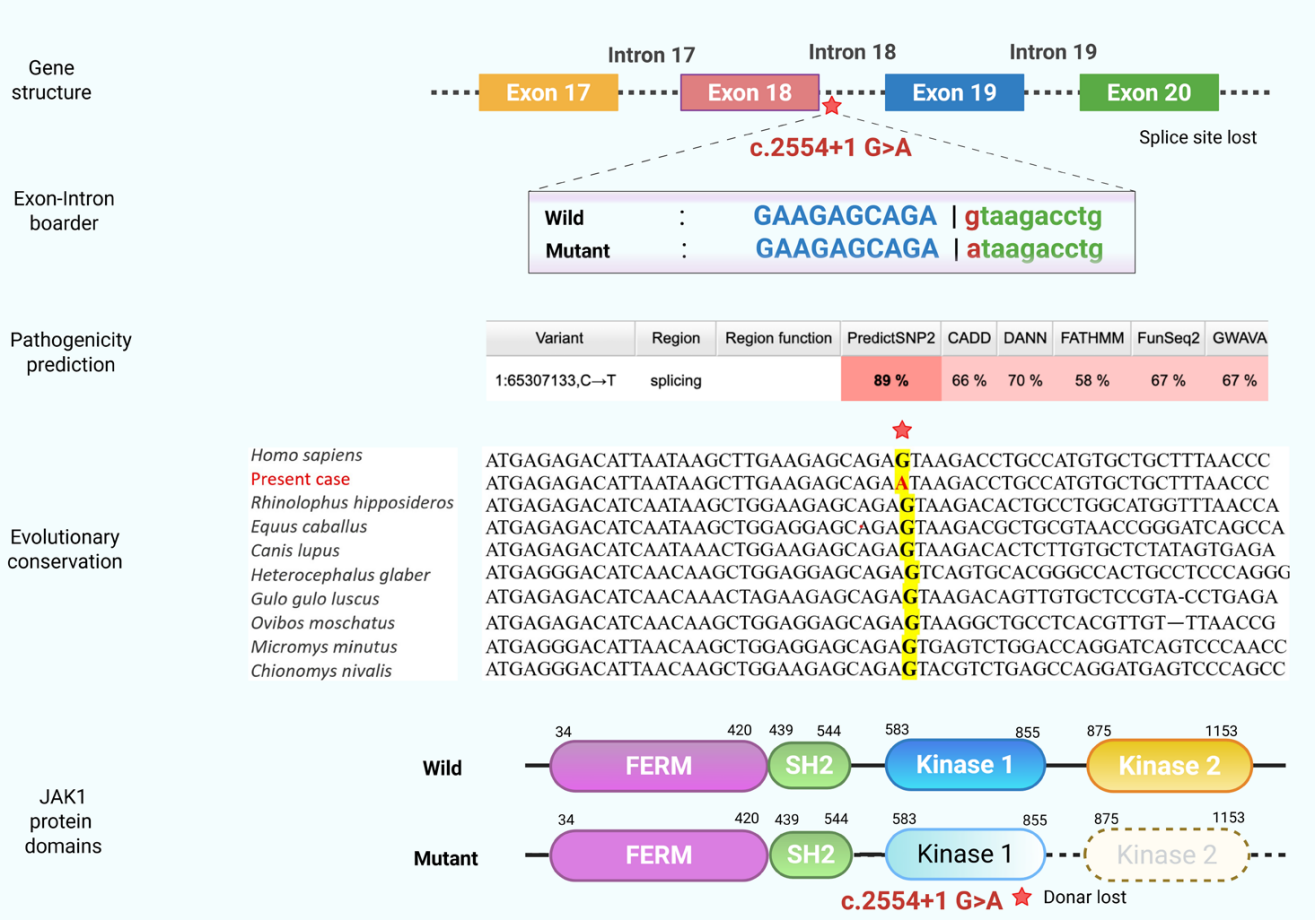
Showing gene structure, location of the mutation, pathogenicity prediction and changes in the JAK 1 protein.

Higher JAK1 expression correlates with better prognosis in breast cancer, particularly in stages I–II (*p* = 0.038) and III–IV (*p* = 0.013) [12]. Conversely, low JAK1 expression may worsen outcomes. The IL‐6/JAK/STAT3 pathway, frequently activated in breast cancer, promotes metastasis by inhibiting apoptosis and enhancing tumour growth by crosstalk with EGFR and AKT pathways (Figure 2) [13]. IL‐6 binds IL‐6Rα, forming a complex with gp130, which activates JAKs and STAT3 via classical, trans‐signalling, or trans‐presentation mechanisms [14, 15, 16]. The IL‐6/JAK/STAT3 Feed‐Forward Loop leads to tumour progression and metastasis [17]. Though it was not required in this case, targeting the IL‐6/JAK/STAT3 pathway is a promising therapeutic strategy. Inhibitors like ruxolitinib (JAK1/2 inhibitor) are under investigation for breast cancer, though resistance mechanisms limit efficacy. Baricitinib and upadacitinib, approved for autoimmune diseases, show potential synergy with chemotherapy in other cancers but require further study in breast cancer.

**FIGURE 2:**
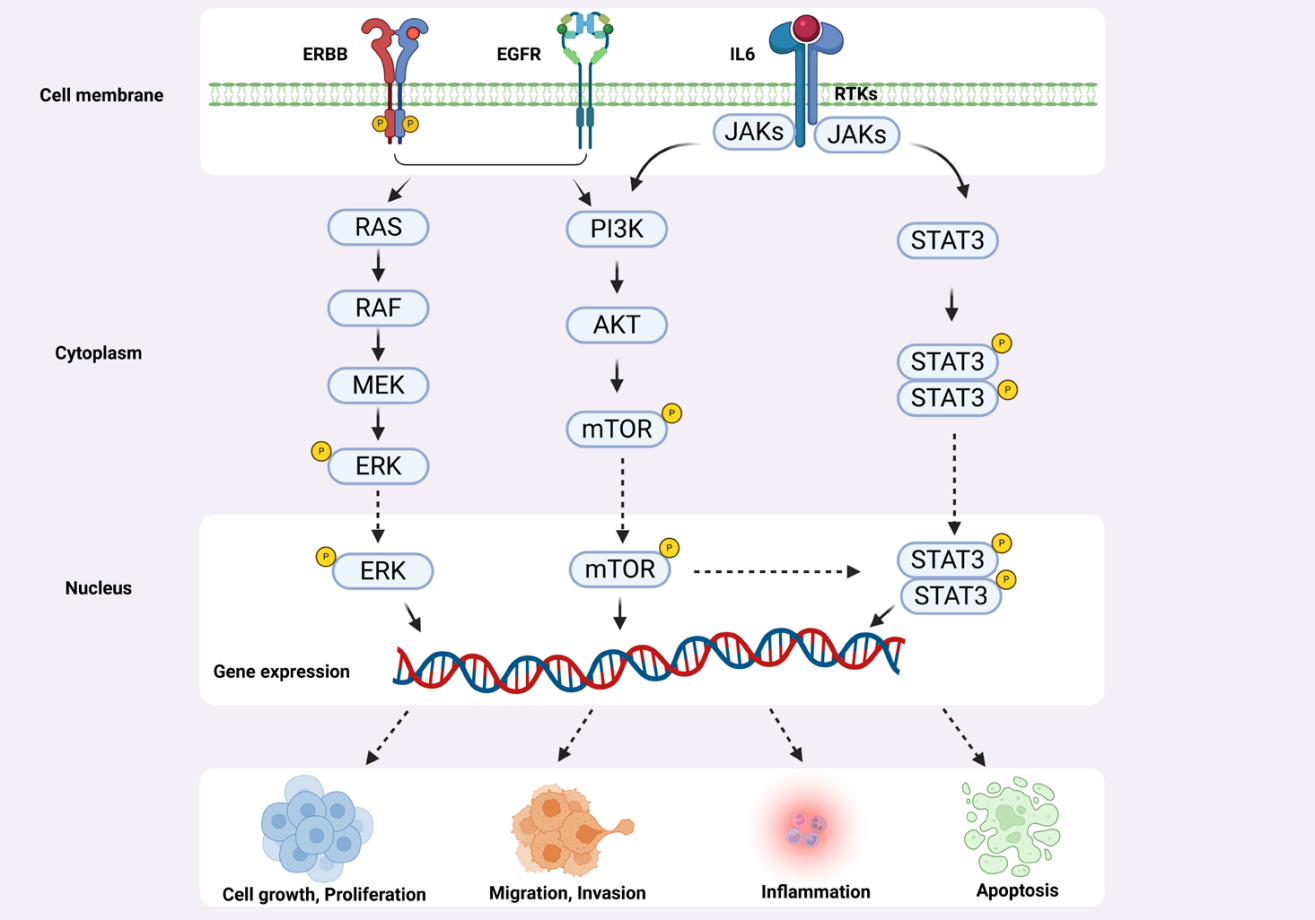
JAK/STAT signalling pathway and cross talk with EGFR and IL6 pathways.

This case highlights a novel JAK1 mutation in IDC, identified through NGS, which may disrupt JAK/STAT signalling and contribute to tumour progression. The patient's favourable outcome underscores the value of comprehensive treatment and genetic profiling. Targeting the JAK/STAT pathway with inhibitors offers potential for personalised therapies in breast cancer.

## Author Contributions

P.K.: literature search, preparation of manuscript, and overall coordination. M.R.: literature search, describing the crosstalk pathway. M.P.: concept and design and final editing. All authors have read and approved the final manuscript.

## Consent

Written informed consent was obtained from the patient for publication of this case.

## Conflicts of Interest

The authors declare no conflicts of interest.

## Supporting information


**Data S1:** MutationTaster—Results JAK1.

## Data Availability

The authors have nothing to report.
