# Supplementary material for: A Novel Jak1 Gene Mutation in Invasive Breast Carcinoma
Source: J Cell Mol Med. 2025 Oct 26;29(20):e70894. doi: 10.1111/jcmm.70894 (PMC12554786; doi:10.1111/jcmm.70894)
Supplement: Supplementary file 1 — Data S1: MutationTaster—Results JAK1. [file JCMM-29-e70894-s001.pdf]

mutation t@sting

NEVER press reload or F5 - unless you want to start from the very beginning.

Input seems to be ok - now mapping the variant to the different transcripts...

Querying Taster for transcript #1: ENST00000342505

MT speed 0.02 s - this script 2.246515 s

Transcript summary:

Permalink

**Transcript**	ENST00000342505
**Gene symbol**	JAK1
**Prediction**	Deleterious
**Tree vote**	88|12
**Model**	without_aae
**Prediction problem**	
**Splice site change**	No
**Known ClinVar disease mutation**	
**Potential ClinVar disease mutation**	
**Amino acid changes**	
**Variant type**	Single base exchange

**dbSNP ID**	
**Protein length**	N/A
**Features at a glance**	Splice site lost

Variant: **1:65307133C>T_1_ENST00000342505** [Back to summary table](#)

Prediction: **Deleterious** [Permalink](#)

Summary:

- **Splice site lost**

- Model: without_aae
- Tree vote: 88|12 (del | benign) ?

**Analysed issue**	**Analysis result**
Phys. location	chr1:65307133C>T show variant in all transcripts IGV
Gene symbol	JAK1
ExAC LOF metrics	LOF: 0.99, misssense: 4.48, synonymous: 0.85
Ensembl transcript ID	ENST00000342505.4
Genbank transcript ID	NM_002227 (exact from MANE)
UniProt peptide	P23458
Variant type	Single base exchange
Gene region	intron
DNA changes	c.2554+1G>A<br>g.125055G>A

Analysed issue	Analysis result
AA changes	N/A
Frameshift	No
Length of protein	N/A
Known variant	Variant was not found in ExAC, 1000G, or gnomAD.
Phylogenetic conservation	*PhyloP* *PhastCons*<br>(flanking) 2.969 1<br>3.713 1<br>(flanking) 2.969 1
Splice sites	alteration within used splice site, likely to disturb normal splicing<br>effect: Donor lost; gDNA position: 125054; score: wt: 0.00 / mu: 0.00; detection sequence: -; exon-intron border: wt: GAAGAGCAGA|gtaagacctg, mu: GAAGAGCAGA|ataagacctg
Distance from splice site	1
Kozak consensus sequence altered?	N/A
poly(A) signal	N/A
Protein conservation	N/A
Protein features	N/A
AA sequence altered	N/A
Chromosome	1
Strand	-1
Original gDNA sequence snippet	TAATAAGCTTGAAGAGCAGA**G**TAAGACCTGCCATGTGCTGC
Altered gDNA sequence snippet	TAATAAGCTTGAAGAGCAGA**A**TAAGACCTGCCATGTGCTGC
Original cDNA sequence snippet	N/A
Altered cDNA sequence snippet	N/A
Wildtype AA sequence	MQYLNIKEDC NAMAFCAKMR SSKKTEVNLE APEPGVEVIF YLSDREPLRL GSGEYTAEEL<br>CIRAAQACRI SPLCHNLFAL YDENTKLWYA PNRTITVDDK MSLRLHYRMR FYFTNWHGTN<br>DNEQSVWRHS PKKQKNGYEK KKIPDATPLL DASSLEYLFA QGQYDLVKCL APIRDPKTEQ

Analysed issue	Analysis result
	DGHDIENECL GMAVLAISHY AMMKKMQLPE LPKDISYKRY IPETLNKSIR QRNLLTRMRI<br>NNVFKDFLKE FNNKTICDSS VSTHDLKVKY LATLETLTKH YGAEIFETSM LLISSENEMN<br>WFHSNDGGNV LYYEVMVTGN LGIQWRHKPN VVSVEKEKNK LKRKKLENKH KKDEEKNKIR<br>EEWNNFSYFP EITHIVIKES VVSINKQDNK KMELKLSSHE EALSFVSLVD GYFRLTADAH<br>HYLCTDVAPP LIVHNIQNGC HGPICTEYAI NKLRQEGSEE GMYVLRWSCT DFDNILMTVT<br>CFEKSEQVQG AQKQFKNFQI EVQKGRYSLH GSDRSFPSLG DLMSHLKKQI LRTDNISFML<br>KRCCQPKPRE ISNLLVATKK AQEWQPVYPM SQLSFDRILK KDLVQGEHLG RGTRTHIYSG<br>TLMDYKDDEG TSEEKKIKVI LKVLDPSHRD ISLAFFEAAS MMRQVSHKHI VYLYGVCVRD<br>VENIMVEEFV EGGPLDLFMH RKSDVLTTPW KFKVAKQLAS ALSYLEDKDL VHGNVCTKNL<br>LLAREGIDSE CGPFIKLSDP GIPITVLSRQ ECIERIPWIA PECVEDSKNL SVAADKWSFG<br>TTLWEICYNG EIPLKDKTLI EKERFYESRC RPVTPSCKEL ADLMTRCMNY DPNQRPFFRA<br>IMRDINKLEE QNPDIVSEKK PATEVDPTHF EKRFLKRIRD LGEGHFGKVE LCRYDPEGDN<br>TGEQVAVKSL KPESGGNHIA DLKKEIEILR NLYHENIVKY KGICTEDGGN GIKLIMEFLP<br>SGSLKEYLPK NKNKINLKQQ LKYAVQICKG MDYLGSRQYV HRDLAARNVL VESEHQVKIG<br>DFGLTKAIET DKEYYTVKDD RDSPVFWYAP ECLMQSKFYI ASDVWSFGVT LHELLTYCDS<br>DSSPMALFLK MIGPTHGQMT VTRLVNTLKE GKRLPCPPNC PDEVYQLMRK CWEFQPSNRT<br>SFQNLIEGFE ALLK*
Mutated AA sequence	
Position of stopcodon in wt / mu CDS	N/A
Position (AA) of stopcodon in wt / mu AA sequence	N/A
Position of stopcodon in wt / mu cDNA	N/A
Position of start ATG in wt / mu cDNA	250 / 250
Last intron/exon boundary	3618
Theoretical NMD boundary in CDS	3318
Length of CDS	3465
Coding sequence (CDS) position	N/A
cDNA position	N/A
gDNA position	125055
Chromosomal position	65307133
Speed	0.02 s

All positions are in basepairs (bp) if not explicitly stated differently. cDNA/gDNA/chromosomal position: Ins/del are shown as 'last normal base / first normal base'.
AA/aa: amino acid; CDS: coding sequence; mu: mutated; NMD: nonsense-mediated mRNA decay; nt: nucleotide; wt: wildtype; TGP: 1000 Genomes Project

Back to summary table
